# Voronoia 4-ever

**DOI:** 10.1093/nar/gkab466

**Published:** 2021-06-09

**Authors:** Rene Staritzbichler, Nikola Ristic, Andrean Goede, Robert Preissner, Peter W Hildebrand

**Affiliations:** University of Leipzig, Institute of Medical Physics and Biophysics, Leipzig, Germany; University of Leipzig, Institute of Medical Physics and Biophysics, Leipzig, Germany; Charité - Universitätsmedizin Berlin, corporate member of Freie Universität Berlin and Humboldt-Universität zu Berlin, Institute of Physiology, Structural Bioinformatics Group, Berlin 10117, Germany; Charité - Universitätsmedizin Berlin, corporate member of Freie Universität Berlin and Humboldt-Universität zu Berlin, Institute of Physiology, Structural Bioinformatics Group, Berlin 10117, Germany; Charité –Universitätsmedizin Berlin, corporate member of Freie Universität Berlin and Humboldt-Universität zu Berlin, Department of Information Technology, Science IT, Charitéplatz 1, 10117 Berlin, Germany; University of Leipzig, Institute of Medical Physics and Biophysics, Leipzig, Germany; Charité –Universitätsmedizin Berlin, corporate member of Freie Universität Berlin and Humboldt-Universität zu Berlin, Institute of Medical Physics and Biophysics, Charitéplatz 1, 10117 Berlin, Germany; Berlin Institute of Health at Charité – Universitätsmedizin Berlin, Charitéplatz 1, 10117 Berlin, Germany

## Abstract

We present an updated version of the Voronoia service that enables fully automated analysis of the atomic packing density of macromolecules. Voronoia combines previous efforts to analyse 3D protein and RNA structures into a single service, combined with state-of-the-art online visualization. Voronoia uses the Voronoi cell method to calculate the free space between neighbouring atoms to estimate van der Waals interactions. Compared to other methods that derive van der Waals interactions by calculating solvent-free surfaces, it explicitly considers volume or packing defects. Large internal voids refer either to water molecules or ions unresolved by X-ray crystallography or cryo-EM, cryptic ligand binding pockets, or parts of a structural model that require further refinement. Voronoia is, therefore mainly used for functional analyses of 3D structures and quality assessments of structural models. Voronoia 4-ever updates the database of precomputed packing densities of PDB entries, allows uploading multiple structures, adds new filter options and facilitates direct access to the results through intuitive display with the NGL viewer. Voronoia is available at: htttp://proteinformatics.org/voronoia.

## Graphical Abstract

**Graphical Abstract:**
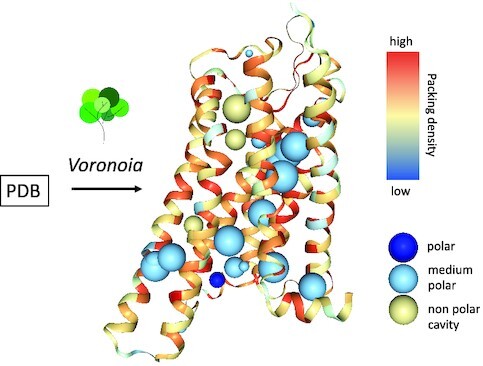
Voronoia analyses and visualises internal packing densities and the polarity of cavities.

## INTRODUCTION

Macromolecules are built from atoms that interact to form 3D structures. Major forces driving folding of proteins is the hydrophobic effect, hydrogen bonding, along with Coulombic interactions and van der Waals interactions. The latter is a distance-dependent force that plays a fundamental role in stabilizing protein structures where multitudes of such interactions are present (1). The atomic packing of protein atoms is thus considered an important indicator of stability, function, folding and unfolding (2,3). The major packing force for water soluble proteins is the hydrophobic effect driven by stronger interactions between water molecules via hydrogen bonding and their entropy gain if a protein is expulsed to its own phase. While optimal packing can generally lead to high stability, packing defects may facilitate the functionally important conformational changes (4,5). In membrane proteins, the absence of the hydrophobic effect as the main driving force is not compensated by the optimization of van der Waals interactions, as originally expected, and may therefore lead to overall lower internal packing densities associated with high structural flexibilities (4). Stability and function are accordingly both properties of a protein, which had to be balanced against each other in the course of evolution (6). Proteins are, thus, not perfectly packed but rather contain a relatively high number of packing defects such as voids or pockets (7–9). Analysis of packing and the search for packing defects, therefore, provide important indicators for understanding the stability and function of macromolecules. Molecular packing densities have been used to estimate thermostability (10,11), calculate the intrinsic compressibility of proteins (2), find ligand binding pockets (12), predict the effects of mutations (13,14) or more generally to design proteins with increased stability (15). In addition, the calculation of packing densities finds use to identify flexible regions in proteins (16), maybe instrumental to interpret high pressure analysis data (17) and more generally, as an indication of unresolved water molecules in tertiary structures or to estimate the quality of protein models (18).

A number of methods were developed to assess the spatial proximity of atoms in 3D, initially by assigning space to Voronoi polyhedra (19), before the introduction of atomic radii allowed the Voronoi method to be applied to proteins (20). This method was further improved by the Voronoi cell method, using curved rather than planar interfaces and treating atoms at cavities and at the protein surface separately (21). Other methods with similar objectives have been developed, such as the Alpha Shape method (15), which models atoms as self-inflating spheres, and the Occluded Surface method (22), which estimates packing by calculating the length of rays protruding from an atom. ProteinVolume generates the molecular surface of a protein and uses a flood-fill algorithm to calculate the individual components of the molecular surface volume, van der Waals and intramolecular void volumes (23).

Voronoia uses the Voronoi cell method and is implemented as a web tool equipped with a convenient user interface for evaluation and visualization of packing densities, accessible to non-programmers. It explicitly considers volume or packing defects to derive van der Waals interactions. Large voids refer to functional important sides within the molecule such as buried ions or water molecules or cryptic ligand binding pockets. Unlike prominent tools for predicting ligand binding pockets or measuring protein channels (24–26), it evaluates only buried cavities, which are not accessible to the bulk phase. Compared to previous versions (27,28), the database of precomputed packing densities of PDB entries has been updated. In addition, an intuitive display is used through the NGL Viewer (29), which provides comprehensive molecular visualization through a graphical user interface directly in the browser. New options for faster calculations and uploading multiple structures have been added, as well as new options for customizing the upload or filtering the results. Cavities can now be distinguished, both by their content and by their neighboring atoms.

## METHODS AND DESCRIPTION

### Voronoi cell method

The original Voronoi method (30) partitions space between points by constructing planes at equidistance from each pair of adjacent points. In 3D space, the constructed planes intersect to form polyhedral volumes enclosing these points. In the context of macromolecules, the polyhedral volumes are created around the positions of atoms to estimate the volume occupied by each atom. In addition, the planes are constructed at a distance weighted by the radii of the atoms vdW to account for different atom sizes.

To calculate molecular packing densities, we applied the Voronoi cell method, which, unlike the original Voronoi method, uses hyperbolic surfaces instead of planes to assign atomic volumes (9). In this method, the solvent-excluded volume of a 3D structure with a probe radius of 1.4 Å is first calculated to distinguish between buried and exposed atoms. Two different volumes are calculated for each atom. *V*_vdW_ is the volume assigned to each atom by the Voronoi cell method, but is only within the _vd_W sphere of the atom. *V*_se_, is the remaining volume, excluded from the solvent, assigned to each atom. From these two volumes, the packing density (PD) for each atom is calculated as

$$\begin{equation*}{\rm PD} = {{V}_{\rm vdW}}/\left( {{{V}_{\rm vdW}} + {{V}_{\rm se}}} \right).\end{equation*}$$

The calculated value of the packing density depends directly on the set of atomic radii used. Voronoia uses the ProtOr (9) and NucProt (31) sets of atomic radii for proteins and nucleotides, respectively, which were determined analytically from reference structures. The atom types of RNA and proteins are grouped according to their chemical nature, valence, and number of hydrogen atoms bound. To determine the vdW radii of these atomic groups, the contacts between each atomic group were analyzed and an average radius was calculated. Internal voids are determined analytically from the structures by Delaunay triangulation (30). Internal voids are defined as a buried space within the structure large enough to accommodate at least one water-size probe with a radius of 1.4 Å.

**Figure 1.**
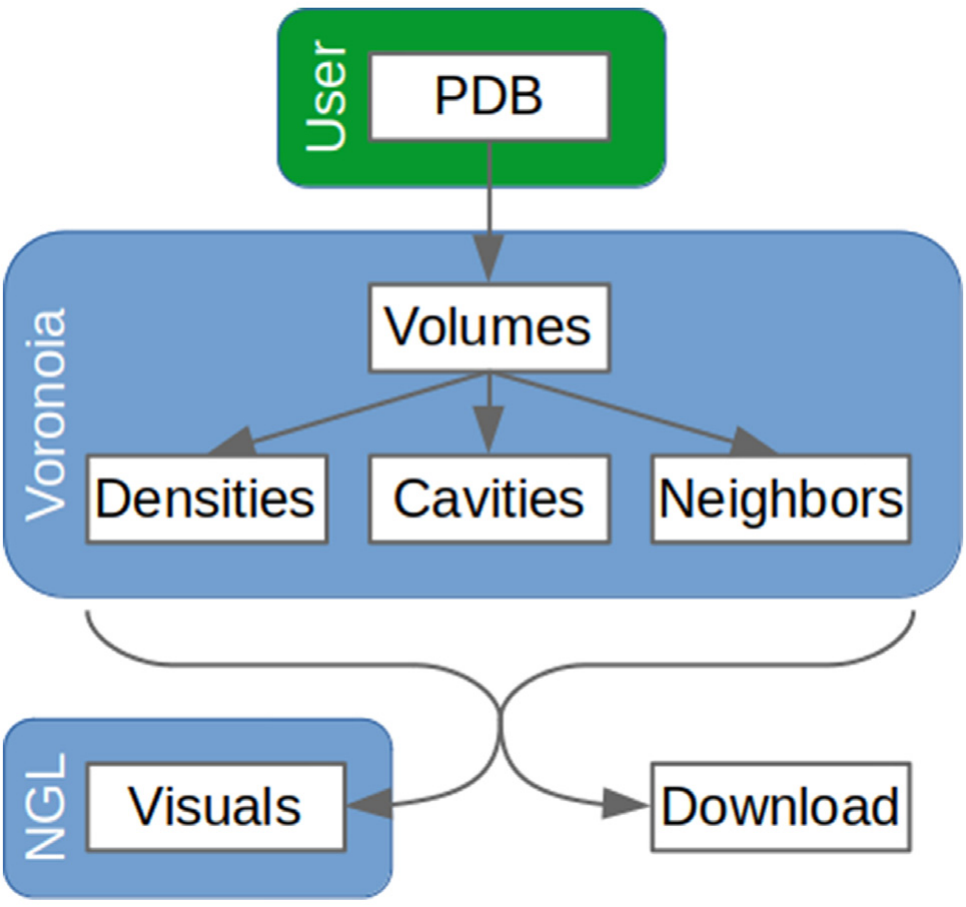
The workflow of the Voronoia server. The user uploads or selects a PDB, for which Voronoia then calculates atomic volumes and packing densities. Cavities with adjacent residues are also identified. The results are immediately visualized with the NGL viewer and are also available for download.

The new implementation of the Voronoia server provides powerful visualizations of both packing densities and size and hydrophobicity of cavities of macromolecules (Figure 1). Earlier separate versions for protein (27) and RNA (28) have been merged and extended with the high-level visualizations of the NGL viewer (29). Leveraging the features of modern web browsers, the ‘NGL Viewer’ supplies fast, hardware-accelerated molecular graphics without the need to install specialized software. The viewer thus offers general-purpose molecular visualization to simplify access 3D structural data for life scientists.

### Server

**Figure 2.**
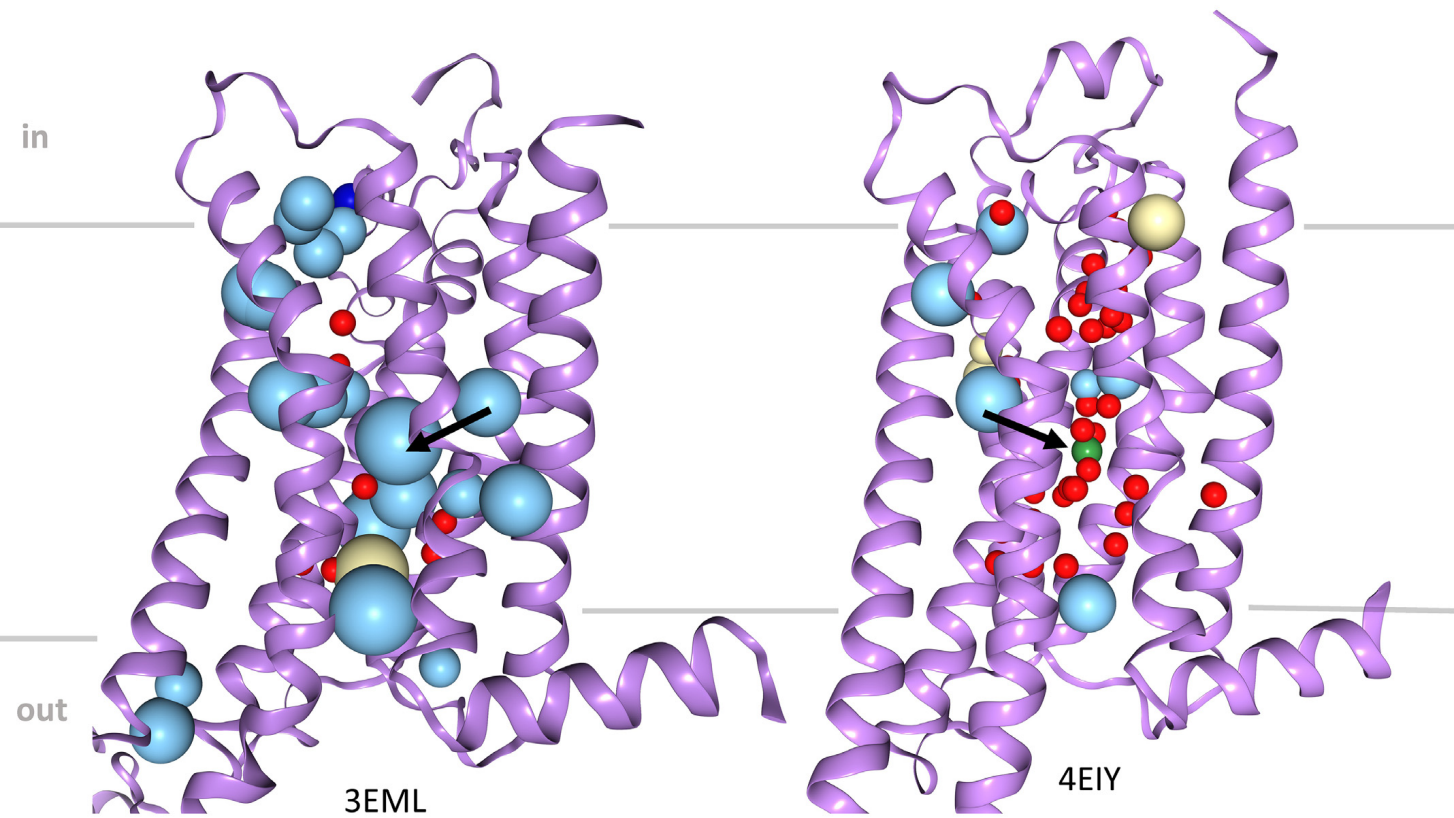
Visualization of large packing defects in X-ray structures of the A2A receptor resolved at different resolutions. Analysis of internal packing densities reveals that the huge cavities in the centre of the A2A receptor structure resolved at 2.3 Å resolution (PDB-entry code: 3EML, left) actually refer to a big cluster of internal water and a sodium ion observed in the corresponding structure resolved at 1.8 Å (4EIY). It is noteworthy that no cavity is found in the lower resolution structure (left) for the absent water cluster at the extracellular site of the receptor near the ligand binding pocket (right), as this cavity is water accessible and thus not internal. Water is shown as a red sphere, potassium as a green sphere, hydrophobic cavities are shown as yellow spheres, and mid-polar cavities in light blue.

**Figure 3.**
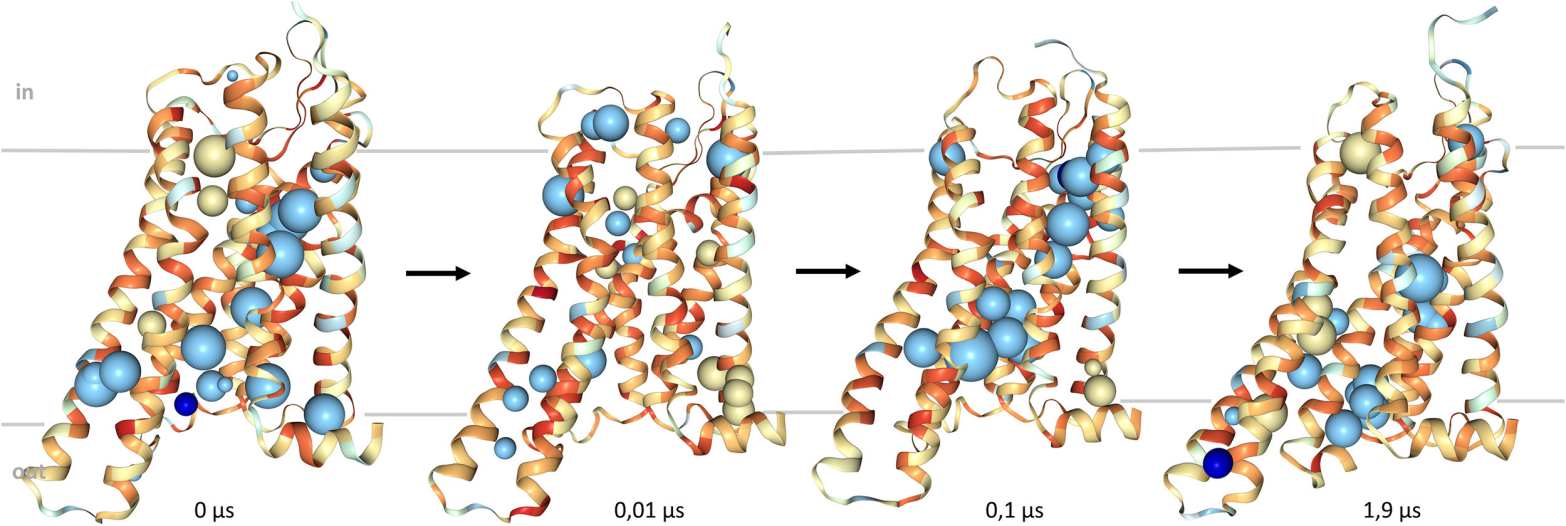
Snapshots in a nearly logarithmic time scale from a Molecular Dynamics simulation of the X-ray structure of the active delta opioid receptor (PDB-entry code: 6pt2) of 1.9 μs without ligand (apostate) and without G protein embedded in a solvated POPC bilayer. Water and heteroatoms were removed before calculating packing densities to highlight the size of internal cavities at one spot. The size and arrangement of internal cavities change significantly as the receptor undergoes different substates. It is noteworthy that an internal cavity that becomes accessible to bulk water is no longer indicated as such. Hydrophobic, intermediate polar, and polar cavities are shown as spheres coloured yellow, light blue and dark blue, respectively. Changes in packing densities (yellow = low, red = high values) indicate that local compressibility, together with shifts in water-binding sites (blue cavities), promotes protein dynamics and ultimately, function.

The server offers two distinct modes of usage. Users can upload their own models for analysis, which is especially valuable for quality assessment in the context of applications such as structure modeling and macromolecular design or for indications of functionally important buried sides such as ions or water molecules (Figure 2). Among many other possible scenarios, snapshots from Molecular Dynamics (MD) simulation trajectories can be investigated for stability (Figure 3). For that purpose, a batch upload, up to 100 pdbs with maximum 10MB has been enabled.

The calculation speed is determined by the resolution of the underlying grid-based method. With the new update, the default accuracy has been set to 0.4 Å for the underlying grid. An option for an accuracy of 0.1 Å is still available (‘high accuracy’). The default setting of 0.4 Å was chosen because it gives comparable results but requires significantly less computation time. The speedup is a factor of 15 for small molecules and a factor of 4 for large molecules, while the accuracy is reduced by only 0.6% (standard deviation 0.5%) for volumes and 0.25% (standard deviation 0.2%) for density. A further reduction or enlargement of the grid, on the other hand, does not make sense, since the gain in accuracy would then be associated with a disproportionately high increase in computing time or the decrease in computing time with a comparatively high loss in accuracy, respectively. Finally, there is a choice between keeping all waters, keeping only the internal waters (default), or deleting all water.

In the first step of the analysis, the molecular volume is assigned to the individual atoms. The atoms are represented by spheres with radii from ProtOr and NucProt (22) sets (9,31). The resulting volume is smoothed by a rolling sphere approach. The Voronoi cell method then assigns the smoothed volume to the atoms that form it (21). The packing density is calculated from these volumes. The next step is the detection of cavities or voids hidden in the molecular volumes. The residues forming the cavity are determined and both the size of the cavity and the average hydrophobicity of the neighboring residues are calculated. Hetero atoms are displayed in a slightly transparent ‘ball and stick’ representation in order to distinguish them from cavity forming side-chains.

The results of this analysis are stored in three independent PDB-formatted files that form the basis for visualization and are also available for download. First, the entire molecule is stored with the packing density of each residue in the *b*-factor entry. Second, the voids are stored as pseudo-atoms with the radius in the *b*-factor and the average hydrophobicity of the residues forming them in the occupancy column. The third PDB file contains only cavity-forming residues whose hydrophobicity is stored in the *b*-factor field.

For experimentally determined structures deposited in the PDB the analysis is pre-calculated to promote fast insight into the packing patterns of protein, DNA, RNA and their complexes. The pre-calculated analysis of PDB entries is updated on a weekly basis. The server is implemented in Flask, a Python framework, uses the Materialize framework in the front end, and allows HTTPS access. The NGL viewer is implemented in Javascript, so no additional plugins are needed for visualization. The volume calculation is written in Delphi and requires Wine to run on Linux. Other calculations are implemented in Python. Downloads are provided on the website.

### Usage

Using the server is quite simple and requires only a few manageable steps. Tutorials, both text and video, are available (https://proteinformatics.uni-leipzig.de/voronoia/tutorial). Users can either select ‘submit’ to upload their own models, or ‘browse PDB’ to search for PDB entries. When selecting ‘browse PDB’, only the PDB ID needs to be specified. Currently 194 000 entries are available. In the ‘submit’ area, a form opens where a file in PDB format can be uploaded, either by clicking on the ‘upload a PDB’ button or by dragging and dropping a file onto the highlighted field. A sample input can be selected.

Once the PDB is selected, the actual submission page opens where a unique tag must be either manually entered or randomly created by clicking the button next to the field. Optionally, an email address can be entered to receive notification that the calculation is complete. Since the job may take several minutes to complete, a status page will open while waiting. A link to the results page is printed and can be copied if the connection to the server is lost.

The results page contains an NGL window, a download button and another button to enter full screen mode. Three different objects are visualized that refer to the described files. Macromolecules are shown in cartoon representation and colored by the packing density of each residue (Figure 3). Cavities are shown as spheres and colored based on the average hydrophobicity of the neighboring residues. Their size is determined by their constituents. Residues adjacent to cavities are shown in ‘licorice’ representation and are colored according to their hydrophobicity. Hydrophobicities are defined by the biological hydrophobicity scale (32), were residues with values below zero are considered non-polar/hydrophobic, values between 0 and 1.45 are considered ’medium polar’, and values above 1.45 are considered polar/hydrophilic. The reason for the cut-off is the significant gap in hydrophobicity between the ‘medium polar’ and hydrophilic residues in the biological hydrophobicity scale. We chose the average value between the two amino acids next to this gap. Hydrophobic residues are coloured gold/yellow, and hydrophilic residues are coloured blue. The combination of size and overall hydrophobicity makes it easier to distinguish between a water-filled cavity and general packing defects.

The molecules can be rotated, moved, and zoomed. Important information about the atoms is accessible by hovering over them. Likewise, the ID of the holes is output in the same way. The files containing all visualized information are available for download as a zip archive. Additionally, a file explaining the contents and format of the zipped files is included. The results page has a link to a full screen view, which for convenience has options to hide side chains and/or cavities. From the full screen view, one can either navigate back or open the full NGL menu. In addition to the obvious flexibility in the displays, the full menu view allows, for example, the selection of the different cavity types. There are four types of cavities, one is empty, one is empty but has heteroatoms as direct neighbors. The third type is similar to the second, except that it shows the cavity if the heteroatoms were not present. Finally, there are cavities that actually contain heteroatoms. For a more detailed explanation of the features, such as creating publication-level images, see the FAQ section of the server.

### Example output

G protein-coupled receptors (GPCRs) are highly flexible proteins that exist in a variety of different inactive or active conformations (33). GPCRs have been resolved using a variety of techniques and at different resolutions. In high-resolution structures, positions of water molecules and ions become visible that are not resolved at lower resolutions. A prominent example is the A2A receptor, which has been elucidated by X-ray structural analysis to high resolution. Comparison of available structures of inactive states of the A2A receptor at 1.8 Å (PDB entry code: 4EIY) and 2.3 Å resolution immediately reveals the lack of structural detail in the lower resolution structure, evident by a higher number and larger size of cavities. These voids relate mainly to positions of waters that are not resolved at 2.3 Å resolution. Strikingly, there is a cluster of water molecules in the higher resolution structure with a central cluster bearing a sodium ion (Figure 2, black arrow), which led to the idea that GPCR signalling is allosterically regulated by sodium (34). Internal cavities discovered by Voronoia thus point to functionally relevant sites that, in combination with other bioinformatic methods for placing water molecules or ions (35,36), can help to tackle the complex nature of GPCR structures to design novel synthetic allosteric modulators or bitopic ligands that e.g. exploit the sodium ion binding pocket.

Conformational changes of proteins are accompanied by rearrangements of amino acid side chains, often controlled by rearrangements of internal water molecules. MD simulations can be used to observe transitions between different substates. Molecular dynamics simulations show the time-resolved motion of macromolecules with atomic resolution and can be used to visualize dynamics on time scales from picoseconds to milliseconds (37). Voronoia can provide insights into changes in molecular packing of selected sub-states visited along a trajectory. For the simulation, we chose the 3D structure of the active delta opioid receptor in complex with the peptide agonist KGCHM07 (PDB entry code: 6pt2). The example output illustrates that the internal packing of the apostate of the receptor changes significantly during a 1.9 μs molecular dynamics simulation. By calculating the packing densities of representative intermediate states, Voronoia can serve as a tool to analyse how intramolecular packing guides protein dynamics and facilitates conformational transitions. To promote the analysis of different representative states, the updated version of Voronoia allows uploading of zipped structure files and overall faster computation.

## CONCLUSION

The Vorononia web service has been updated to provide instant access to all entries listed in the RCSB PDB, for which packing densities and the position of internal cavities have been precomputed. Previous separate versions for proteins and RNA have been merged and enhanced with the high-level visualizations of the NGL Viewer, which provides universal molecular visualization to simplify access to 3D structural data for life scientists. Many new features, such as batch mode, filtering, cavity selection and performance-related options are provided.

## DATA AVAILABILITY

Voronoia is available as a free web service with tutorial, FAQs and download page, without the need to register or provide any information: http://proteinformatics.org/voronoia. The Voronoia webservice uses the NGL viewer (29).

NGL is an open source collaborative initiative available in the GitHub repository (https://github.com/nglviewer/ngl)
